# Chromosomal assembly of the flat oyster (*Ostrea edulis* L.) genome as a new genetic resource for aquaculture

**DOI:** 10.1111/eva.13462

**Published:** 2022-10-10

**Authors:** Isabelle Boutet, Homère J. Alves Monteiro, Lyam Baudry, Takeshi Takeuchi, Eric Bonnivard, Bernard Billoud, Sarah Farhat, Ricardo Gonzales‐Araya, Benoit Salaun, Ann C. Andersen, Jean‐Yves Toullec, François H. Lallier, Jean‐François Flot, Nadège Guiglielmoni, Ximing Guo, Cui Li, Bassem Allam, Emmanuelle Pales‐Espinosa, Jakob Hemmer‐Hansen, Pierrick Moreau, Martial Marbouty, Romain Koszul, Arnaud Tanguy

**Affiliations:** ^1^ Sorbonne Université, CNRS, UMR 7144 Station Biologique de Roscoff Roscoff France; ^2^ National Institute of Aquatic Resources Technical University of Denmark Silkeborg Denmark; ^3^ Institut Pasteur Unité Régulation Spatiale des Génomes, CNRS Paris France; ^4^ Marine Genomics Unit Okinawa Institute of Science and Technology Graduate University Okinawa Japan; ^5^ Sorbonne Université, CNRS UMR 8227, Station Biologique de Roscoff Roscoff France; ^6^ Marine Animal Disease Laboratory, School of Marine and Atmospheric Sciences Stony Brook University Stony Brook New York USA; ^7^ Centre Régional de la Conchyliculture Bretagne Nord Morlaix France; ^8^ Evolutionary Biology and Ecology Université Libre de Bruxelles Brussels Belgium; ^9^ Haskin Shellfish Research Laboratory, Department of Marine and Coastal Sciences Rutgers University Port Norris New Jersey USA; ^10^ Department of Marine Organism Taxonomy and Phylogeny, Institute of Oceanology Chinese Academy of Sciences Qingdao China

**Keywords:** aquaculture, flat oyster, genome, Martelia, transposable elements

## Abstract

The European flat oyster (*Ostrea edulis* L.) is a native bivalve of the European coasts. Harvest of this species has declined during the last decades because of the appearance of two parasites that have led to the collapse of the stocks and the loss of the natural oyster beds. *O. edulis* has been the subject of numerous studies in population genetics and on the detection of the parasites *Bonamia ostreae* and *Marteilia refringens.* These studies investigated immune responses to these parasites at the molecular and cellular levels. Several genetic improvement programs have been initiated especially for parasite resistance. Within the framework of a European project (PERLE 2) that aims to produce genetic lines of *O. edulis* with hardiness traits (growth, survival, resistance) for the purpose of repopulating natural oyster beds in Brittany and reviving the culture of this species in the foreshore, obtaining a reference genome becomes essential as done recently in many bivalve species of aquaculture interest. Here, we present a chromosome‐level genome assembly and annotation for the European flat oyster, generated by combining PacBio, Illumina, 10X linked, and Hi‐C sequencing. The finished assembly is 887.2 Mb with a scaffold‐N50 of 97.1 Mb scaffolded on the expected 10 pseudochromosomes. Annotation of the genome revealed the presence of 35,962 protein‐coding genes. We analyzed in detail the transposable element (TE) diversity in the flat oyster genome, highlighted some specificities in tRNA and miRNA composition, and provided the first insight into the molecular response of *O. edulis* to *M. refringens*. This genome provides a reference for genomic studies on *O. edulis* to better understand its basic physiology and as a useful resource for genetic breeding in support of aquaculture and natural reef restoration.

## 1 BACKGROUND

The European flat oyster, *Ostrea edulis* (NCBI:txid37623), is the native oyster species in Europe and its distribution ranges from the Norwegian Sea in the north to Morocco in the south, and east through the Mediterranean Sea to the Black Sea. The flat oyster industry has been in decline since the 19th century mainly due to its habitat destruction, over‐exploitation, irregular recruitment, and outbreaks of the parasites *Marteilia refringens* and *Bonamia ostreae* during the 1970s (Smaal et al., 2015). In France, the *O. edulis* production amounts to 1100 tons per year and is mainly located in Brittany. Because of its economic and ecological importance, *O. edulis* is subjected to extensive protection and restoration and has been identified as a priority species by the OSPAR Convention (OSPAR, 2009). Indeed, the flat oyster ecosystem is similar to calcareous biogenic reefs and contributes to substrate stabilization, water filtration, and habitat formation for other species (Beck et al., 2011). Unlike the *Crassostrea* oysters *Crassostrea gigas* or *Crassostrea virginica*, *O. edulis* is a larviparous species with asynchronous hermaphroditism and rhythmic consecutive sexuality. Molecular studies on the physiology of the flat oyster, including basic traits and adaptative response to abiotic parameters, are rare and mainly focused on the study of the response of *O. edulis* to its parasite *B. ostreae* in laboratory experiments (Cocci et al., 2020; Gervais et al., 2019; Ronza et al., 2018). Several programs have aimed to identify quantitative trait loci (QTLs) associated with *B. ostreae* resistance with the use of comparative transcriptomic approaches (Pardo et al., 2016; Ronza et al., 2018) and SNP genotyping (Vera et al., 2019).

Aquaculture is increasingly benefiting from the availability of genomic information, allowing for high‐quality basic and applied genomic research to achieve various objectives such as improving aquaculture production and studying the effect of external factors on the physiology and genetics of the animal species concerned and their pathogens (Bernatchez et al., 2017; Hollenbeck & Johnston, 2018; Potts et al., 2021). While traditional selective breeding has improved disease resistance in some mollusk species when the heritability is high, resistance to some diseases may be also under the control of many small‐effect genes where genomic selection is expected to be more effective (Guo, 2021). To partly resolve these limitations and provide genomic resources for aquaculture breeding, genomes of many bivalves have been sequenced, including the oysters *C. gigas* (Peñaloza et al., 2021; Zhang et al., 2012), *Crassostrea hongkongensis* (Peng et al., 2020), *Crassostrea ariakensis* (Li et al., 2021), *C. virginica* (Modak et al., 2021), and *Saccostrea glomerata* (Powell et al., 2018), the pearl oyster *Pinctada fucata* (Du et al., 2017), the scallops *Patinopecten yessoensis* (Wang et al., 2017) and *Pecten maximus* (Kenny et al., 2020), the blood clam *Scapharca broughtonii* (Bai et al., 2019), the hard‐shelled mussel *Mytilus coruscus* (Yang et al., 2021), and the hard clam *Mercenaria mercenaria* (Farhat et al., 2022; Song et al., 2021). Since 2018, we initiated a selective breeding program (European project PERLE 2) to characterize rusticity parameters, including survival, growth, and resistance to parasites in the flat oyster *O. edulis*. The main objectives of this project are to (1) produce hundreds of full‐sib families tested in different environments (deep water and foreshore), (2) introduce the most performant families in natural oyster beds showing a decline in their population in order to enhance their dynamics and then contribute to their restoration, and (3) specifically select the most performing families on the foreshore in order to reintroduce this mode of culture for *O. edulis*. In the present study, an improved chromosome‐level assembly of *O. edulis* genome was developed through a combination of high coverage Pacific Biosciences (PacBio) long‐read sequencing, 10X chromium library sequencing, accurate Illumina short‐read data and Hi‐C library sequencing. We focused our analyses on the diversity of transposable elements through a comparison with other oyster genomes, highlighted some specificities in noncoding RNA diversity and provided the first RNAseq analysis on *M. refringens*‐infected individuals revealing some original molecular responses.

## 2 MATERIALS AND METHODS

### 2.1 Tissue sampling, RNA isolation, and RNA‐sequencing for genome annotation

Tissues from oyster organs (gill, mantle, digestive gland, adductor muscle, gonads, hemocytes and palp) were sampled from 12 individuals and frozen in liquid nitrogen. Total RNA was extracted using TRIzol Reagent (Invitrogen, Life Technologies) according to the manufacturer's instructions. The RNA quality was checked on agarose gel and quantified using a NanoPhotometer spectrophotometer (Thermo Fisher Scientific). An equal amount of RNA of each tissue from four individuals was pool to generate three libraries per tissue. Libraries were constructed at the McGill Genome Center using NEBNext® Ultra™ Directional RNA Library Prep Kit for Illumina (New England Biolabs) following the manufacturer's recommendations and sequenced by Illumina HiSeq with 2 × 150 cycles (Illumina Inc.).

### 2.2 RNA extraction in *M. refringens‐*infected oyster

Fifty oysters (size: 5–8 cm) were collected at the “banc du Roz” (Bay of Brest, France). Hemolymph was retrieved from the adductor muscle using a syringe, and hemocytes were collected by centrifugation and immediately frozen in liquid nitrogen. On the same individual, a piece of the digestive gland was sampled for DNA extraction and parasite detection by PCR using two specific *M. refringens* primers (Le Roux et al., 1999), and the remaining tissues were frozen in liquid nitrogen. RNA was then extracted from hemocytes and the digestive gland of the six *M. refringens* positive individuals identified by PCR and on six negative individuals using TRIzol Reagent (Invitrogen). The RNA quality was checked on agarose gel and quantified using a NanoPhotometer spectrophotometer (Thermo Fisher Scientific). Two pools containing each an equal amount of RNA from three individuals showing a positive PCR amplification (six individuals in total) and two pools containing each an equal amount of RNA from three individuals showing no PCR amplification (six individuals in total) were generated for RNAseq library construction and sequencing using the same protocol as described above and sequenced by Illumina HiSeq with 2 × 150 cycles (Illumina Inc.).

### 2.3 DNA library preparation and whole genome sequencing

#### 2.3.1 DNA extraction

Genomic DNA used for PacBio, Illumina shotgun, and 10× chromium libraries was extracted from fresh adductor muscle dissected off a single specimen of *O. edulis* (size = 18 cm) collected from the Bay of Morlaix (Brittany, France), using a standard phenol‐chloroform‐isoamyl alcohol (PCI 25:24:1) protocol and ethanol precipitation. DNA quality was checked by electrophoresis on 1% agarose gels and DNA concentration was measured using a Qubit® dsDNA HS Assay Kit in Qubit® 2.0 Fluorometer (Life Technologies).

#### 2.3.2 Shotgun library

Shotgun library was generated at the McGill Genome Center using the NxSeq® AmpFREE Low DNA Library Kit Library Preparation Kit (Lucigen Corp.) according to the manufacturer's recommendations and sequenced by Illumina HiSeqX with 2 × 150 cycles (Illumina Inc.).

#### 2.3.3 Pacbio sequel libraries

The DNA library was prepared following the Pacific Biosciences 20 kb Template Preparation using 7.5 μg of high molecular weight genomic DNA using the SMRTbell Template Prep Kit 1.0 reagents (Pacific Biosciences). The DNA library was size‐selected on a BluePippin system (Sage Science Inc.) using a cutoff range of 12–50 kb. The libraries were sequenced on a PacBio Sequel instrument at a loading concentration (on‐plate) of 6 pM using the diffusion loading protocol, Sequel Sequencing Plate 2.1, SMRT cells 1M v2, and 10 h movies.

#### 2.3.4 10× chromium library

The gDNA was size‐selected on a BluePippin system (Sage Science Inc.) using a cutoff range of 40–80 kb. The 10× Chromium shotgun libraries were prepared following the Chromium Genome Reagent kits v2 User Guide RevB protocol, using the Chromium™ Genome Library & Gel Bead Kit v2, Chromium™ Genome Chip Kit, and Chromium™ i7 Multiplex Kit (10X Genomics Inc.) and sequenced by Illumina HiSeqX with 2 × 150 cycles (Illumina Inc.).

#### 2.3.5 Hi‐C libraries

The Hi‐C library construction protocol was adapted from Lieberman‐Aiden et al. (2009) and Lazar‐Stefanita et al. (2017). Hi‐C library was made from a piece of adductor muscle from another individual from the same population and sequenced on a NextSeq 550 apparatus (2 × 75 bp, paired‐end Illumina NextSeq). Contact maps were generated from reads using the hicstuff pipeline for processing generic 3C data, available at https://github.com/koszullab/hicstuff. The backend uses the bowtie2 (version 2.2.5) aligner run in paired‐end mode (with the following options: ‐‐maxins 5 –very‐sensitive‐local). Alignments with a mapping quality lower than 30 were discarded. The output was in the form of a sparse matrix where each fragment of every chromosome was given a unique identifier and every pair of fragments was given a contact count if it was nonzero. The assembled genome generated by instaGRAAL was polished to remove misassemblies (Baudry et al., 2020). Initial and final assembly metrics (Nx, GC distribution) were obtained using QUAST‐LG. Misassemblies were quantified using QUAST‐LG with the minimap2 aligner in the backend. Ortholog and assembly completeness was computed with BUSCO (v3). The evolution of genome metrics between cycles was obtained using instaGRAAL's own implementation.

#### 2.3.6 Gene prediction

All RNAseq reads obtained from the different organ transcriptomes were quality‐trimmed using Trimmomatic (version 0.36) (Bolger et al., 2014) and mapped to the genome assembly using HISAT2 (Kim et al., 2019). The alignment information was processed to generate genome‐guided transcriptome assembly using Trinity (ver. 2.8.4) (Grabherr et al., 2011) and in parallel, de novo transcriptome assembly was also generated by the Trinity software. The genome‐guided and de novo transcriptome assemblies were processed by the PASA pipeline (ver. 2.3.3) (Haas et al., 2003) to generate a training set for optimizing gene prediction parameters in AUGUSTUS (Stanke et al., 2006). RNAseq reads were also aligned to the genome using STAR (ver. 2.7.1a) (Dobin et al., 2013) to produce hint files of exon and intron information. Then, de novo gene prediction was performed using AUGUSTUS (ver. 2.5.5). The completeness of the predicted gene models was assessed by Benchmarking Universal Single‐Copy Orthologs (BUSCO) using metazoa_odb9 database (Simão et al., 2015). Gene annotation was made using Trinotate (Grabherr et al., 2011).

#### 2.3.7 Noncoding RNA prediction

tRNA genes were predicted using tRNAscan‐SE v1.3.1 (Lowe & Eddy, 1997) with eukaryote parameters, and rRNA with high conservation were predicted by aligning reads to the *Arabidopsis thaliana* template rRNA sequences using BLASTN (Altschul et al., 1997), with an *e*‐value of 10^−5^. Additionally, INFERNAL (Nawrocki & Eddy, 2013) was used to predict miRNA and snRNA genes on the basis of the Rfam database (Griffiths‐Jones, 2005).

### 2.4 Repeat annotation

#### 2.4.1 LTR‐retrotransposons identification

We specifically investigated LTR‐retrotransposons in four genomes of Ostreidae (*O. edulis*, *C. gigas* (GCA_902806645.1), *C. virginica* (GCA_002022765.4), and *S. glomerata* (GCA_003671525.1)) and one genome of a Pteriidae species, the pearl oyster *Pinctada martensii* (GCA_002216045.1) using a detailed and precise pipeline previously customized for *M. mercenaria* (Farhat et al., 2022). To annotate these LTR‐retrotransposons, we first extracted and translated the RT/RNaseH domain from the sequences obtained with LTRHarvest using BLASTx (Camacho et al., 2009) (*e*‐value <10^−5^) against an in‐house database of RT/RNaseH (Thomas‐Bulle et al., 2018) made from Gypsy Database (Llorens et al., 2011). Here, we kept the single sequences and/or a consensus sequence per previously defined cluster and used them in phylogenetic approaches to determine the position of the elements in each clade. Cladistic analyses were performed on amino acid sequences corresponding to the RT/RNaseH domains of the newly characterized sequences and reference elements. Multiple alignments of these protein sequences were performed using MAFFT (Katoh et al., 2018). After manual curation of the alignments, phylogenetic analyses were conducted using neighbor‐joining and the pairwise deletion option of the MEGA 5.2 software (Tamura et al., 2011). Using Topali2.3 (Milne et al., 2009), the best‐fitted substitution model retained was the JTT model with a gamma distribution. Support for individual groups was evaluated with nonparametric bootstrapping using 100 replicates.

#### 2.4.2 Repeated sequences annotation

Repeated sequences were annotated in the five genomes by running RepeatMasker (http://repeatmasker.org) with the default parameter and the script “Concatenate_sequences.py” (Thomas‐Bulle et al., 2018). Two different libraries were used: (i) one with only inserts‐cleaned consensus from each LTR‐retrotransposon cluster detected by LTRHarvest to retrieve putatively missed copies (with corrupted LTRs or deleted); (ii) the second with all other consensus transposable elements obtained with RepeatModeler v2.0.1 (Flynn et al., 2020) using REPBASE, version 2017‐01‐27 (Jurka et al., 2005).

### 2.5 Phylogenetic analysis

To cluster families from protein‐coding genes, proteins from the longest transcripts of each gene from *O. edulis* and other mollusc species, including *Argopecten irradians* (SRP174526), *A. purpuratus* (PRJNA418203), *Aplysia californica* (GCA_000002075.2), *Biomphalaria glabrata* (GCF_000457365.1), *C. gigas* (GCA_902806645.1), *C. virginica* (GCA_002022765.4), *Chlamys farreri* (in MolluskDB), *Elysia chlorotica* (GCA_003991915.1), *Modiolus philippinarum* (GCA_002080025.1), *P. fucata* (PRJNA283019), *Lottia gigantea* (PRJNA175706), *Octopus binaculoides* (GCF_001194135.1), *S. glomerata* (GCA_003671525.1), *Haliotis discus* (PRJNA317403) were collected in GENBANK and data for *Ostrea denselamellosa* and *Ostrea stentina* were specifically generated for this analysis. Orthofinder version 2.4.1 (Emms & Kelly, 2019) was run using all proteomes with default parameters. From Orthofinder results, we used the single‐copy gene OGs (114OGs) and concatenated the proteins per species to generate the species tree using MAFFT online (Katoh et al., 2018) with default parameters. MEGA version 5.2 software was used to construct the phylogenetic tree with a bootstrap of 1000 on a neighbor‐joining method.

### 2.6 Differential expression analysis

RNAseq reads from *M. refringens*‐infected and ‐noninfected libraries were checked for quality issues and adapter content with FastQC 0.11.7 and cleaning for sequencing adapters, trimming of low‐quality bases (minimum mean quality score of 30), and filtering for length were performed with Trimmomatic 3.3 (Bolger et al., 2014). Reads were aligned to the *Roscoff_O.edulis‐V1* genome assembly with Bowtie 2 (Langmead et al., 2019) to generate BAM files that were used to generate expression counts with the IdxStats software (Li et al., 2009). Raw read counts were first normalized using the FPKM method (Mortazavi et al., 2008), and FPKM values below 5 were eliminated when the gene was present in both conditions. Data were log2 transformed and then imported into R 3.5.0 (R Core Team, 2017) for analysis with DESeq2 package (Love et al., 2014). The genes with a threshold of false discovery rate (FDR) < 0.05 and |log2 (fold change)| >1 were defined as differentially expressed genes (DEGs). Functional enrichment analysis for the Gene Ontology term was performed using DAVID software (Huang et al., 2009) using a Benjamini adjusted *p*‐value of .05.

## 3 RESULTS

### 3.1 Genome assembly and annotation

**FIGURE 1:**
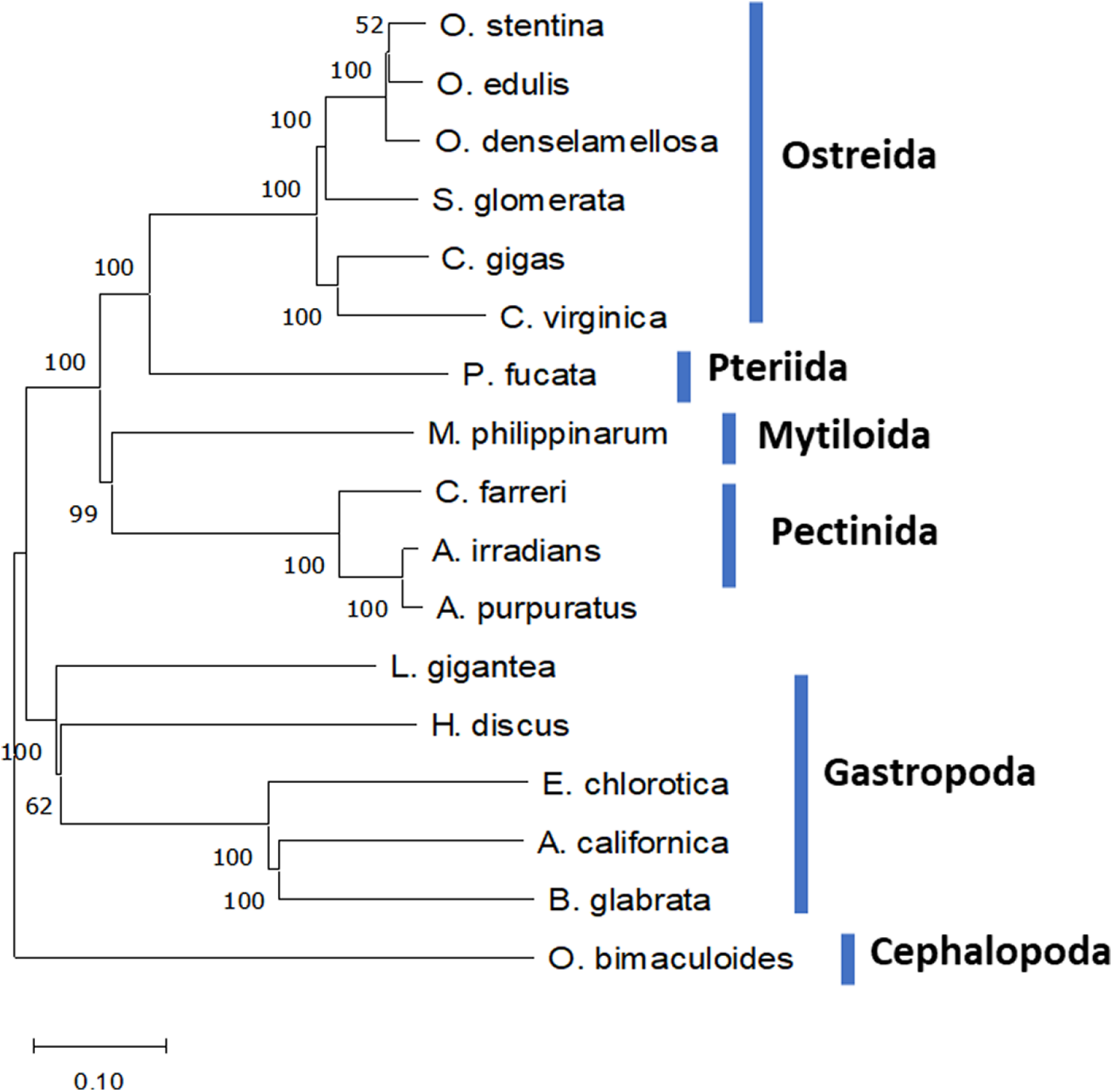
Phylogenetic tree of Mollusca species based on the alignment of 114 OGs. The tree scale is 0.1, and the bootstrap is represented at each node.

PacBio reads (45× raw coverage) and Illumina PE shotgun reads (104× raw coverage) were first assembled into contigs using Masurca hybrid assembler v3.2.8 (Zimin et al., 2013) assuming a 1 Gb genome size estimated by flow cytometry using SYBGREEN labeling (data not shown). Then, the 10× Chromium reads (67× raw coverage) were used by the ARCS v1.0.4 (https://github.com/bcgsc/arcs, v1.0.4) together with LINKs pipeline v1.8.5 (https://github.com/bcgsc/LINKS, v1.8.5) to scaffold and make the Masurca assembly more contiguous. A total of 5417 contigs were obtained for an estimated size of 1028 Mb with an N50 length of 0.94 Mb. HaploMerger2 software (https://github.com/mapleforest/HaploMerger2/releases/; Huang et al., 2017) was used to remove potential duplicated sequences and significantly improved the assembly to 2846 contigs (N50 of 1.62 Mb). A chromosome‐level assembly was then generated using Hi‐C data and InstaGRAAL software. The final assembly of the *Roscoff_O.edulis‐V1* genome was 1018 Mb in size, with the chromosome‐level scaffolds represented in 10 super‐scaffolds corresponding to 887.2 Mb of sequence length and 553 unplaced scaffolds with a total N50 of 97.1 Mb for scaffold lengths. The 10 expected chromosomes are in accordance with the chromosomes previously evidenced in a karyotype analysis (Thiriot‐Quiévreux & Ayraud, 1982) and linkage map (Lallias et al., 2007) (Figure S1). The *Roscoff_O.edulis‐V1* genome shows a GC content of 35.46% and the BUSCO analysis indicates that the gene models include 95.1% complete BUSCOs. A total of 35,962 protein‐coding genes were predicted from which 24,302 genes were functionally annotated (*e*‐value < 10^−3^). The parameters of completeness and main genome features of the assembly are presented in Table 1 and are very close to those obtained in the *OE_Roslin_V1* assembly (Gundappa et al., 2022). A phylogenetic tree confirms that *Roscoff_O.edulis‐V1* clusters with other Ostreidae and has a closer relationship with the other two Chinese *Ostrea* species (Figure 1).

**TABLE 1:** Genome assembly statistics for *Roscoff_O.edulis‐V1* genome.

Metric	Value	Number of genes
10‐chrom assembly size (bp)	887,207,661	
Scaffold 1 (bp)	117,936,894	4971
Scaffold 2 (bp)	111,702,126	4882
Scaffold 3 (bp)	97,982,138	4282
Scaffold 4 (bp)	97,658,951	4001
Scaffold 5 (bp)	97,091,390	4170
Scaffold 6 (bp)	95,771,753	3908
Scaffold 7 (bp)	90,095,513	3357
Scaffold 8 (bp)	74,472,875	2356
Scaffold 9 (bp)	53,076,851	2190
Scaffold 10 (bp)	51,419,170	1846
GC content (%)	35.46	
Complete BUSCOs* (C)	931 (95.1%)	
Complete and single‐copy BUSCOs (S)	911 (93.1%)	
Complete and duplicated BUSCOs (D)	20 (2.0%)	
Protein‐coding genes number	35,963	
mean transcript length	13,916	
mean cds length	1626	
mean exons per transcript	7.2	
mean introns per transcript	5.6	

### 3.2 RNA prediction

Detection of miRNA using INFERNAL and Rfam database allowed the identification of 126 families of miRNAs (*e*‐value <10^−5^) in the *Roscoff_O.edulis‐V1* genome. A large variation in the copy number is also detected among miRNA families and a total of 99 miRNA families contains more than one paralog. The mir‐148 family is the most represented family with 219 copies followed by the mir‐821 family with 106 copies and 23 families exhibiting more than 10 copies (Table S1A). The distribution of the 25 most represented miRNA families shows a relatively homogeneous distribution along the chromosomes with a higher frequency observed in chromosomes 5, 6, 7, 8, and 9. Most of the conserved miRNA families identified in other mollusc species (let‐7, lin‐4, miR‐2, miR‐29, miR‐87, miR‐184, miR‐8, miR‐10, miR‐15, miR‐17) are present in *Roscoff_O.edulis‐V1* genome (Table S1B). The number of miRNA families identified in *O. edulis* (128) are similar to what is detected in *C. gigas* (120 families), *C. virginica* (121 families), or *P. fucata* (126 families) and many of the miRNA classically found in molluscs are present. We performed the same analysis on *C. virginica* and *M. mercenaria* genomes to identify enrichments in miRNA families in *O. edulis*. No miRNA family appears to be specific to *O. edulis* when comparing with *C. virginica*, but *O. edulis* presents a globally higher number of copies for the most represented families (Table 2). By contrast, the diversity of miRNAs in *M. mercenaria* appears to be much lower and some families appear to be specific to oysters such as mir‐148, mir‐1027, mir‐1803, and mir‐355. Only mir‐1253 is present in *M. mercenaria* and absent in oyster genomes (Table 2).

**TABLE 2:** Numbers of copies for the 14 most represented miRNA in the genomes of *Ostrea edulis*, *Mercenaria mercenaria*, and *Crassostrea virginica* (miRNA annotation according to RFam database).

miRNA code in RFam	*O. edulis*	*C. virginica*	*M. mercenaria*
Mir‐1253	0	0	29
Mir‐148	219	157	0
Mir‐1222	24	20	2
Mir‐1027	76	62	0
Mir‐1023	84	54	6
Let‐7	17	17	2
Mir‐1803	28	13	0
Mir‐2118	43	27	3
Mir‐598	6	3	26
Mir‐1122	65	48	12
Mir807	23	19	4
Mir‐944	50	47	4
Mir‐355	31	22	0
Mir‐2118	41	27	3

A total of 1985 annotated tRNAs representing the 20 amino acids, three copies of the 5.8S rRNA, six copies of the LSU‐rRNA, and six copies of SSU‐rRNA, but 54 copies of the 5S‐rRNA have been annotated (Table 3). Two tRNA families are over‐represented with 1019 copies of the tRNA‐Ser from which 907 correspond to anticodon AGA and 339 copies of the tRNA‐Thr from which 282 copies correspond to AGU. tRNA‐Ser gene distribution exhibits a homogenous repartition in the genome (Figure S2). The pairwise alignment scores confirm that the tRNA‐Ser motif is not included in a repeated element that could explain the high number of copies observed. The comparison of tRNA composition in *M. mercenaria* and *C. virginica* genomes using the same parameters showed that enrichment in tRNA‐Ser was only detected in *O. edulis*, but a higher content in tRNA‐Thr (791 copies) and tRNA‐Ala (465 copies) was detected in *M. mercenaria*. The density of tRNA is higher in *O. edulis* (2.275 tRNA/Mb) than in *M. mercenaria* (1.18 tRNA/Mb) or in *C. virginica* (0.575 tRNA/Mb). Comparison with available *C. gigas* tRNA composition showed a similar pattern with 641 tRNA genes and a density of 1.08 tRNA/Mb for the Pacific oyster.

**TABLE 3:** Numbers of tRNA and rRNA in the genome of *Ostrea edulis*, *Mercenaria mercenaria*, and *Crassostrea virginica*.

	*O. edulis*	*M. mercenaria*	*C. virginica*
tRNA_Ser	1019	96	53
tRNA_Phe	42	25	11
tRNA_Asp	43	26	37
tRNA_Glu	31	20	56
tRNA_iMet	42	30	36
tRNA_Tyr	30	24	17
tRNA_Leu	44	51	60
tRNA_Lys	37	45	62
tRNA_Gln	39	48	21
tRNA_Ile	34	43	26
tRNA_Cys	20	26	10
tRNA_Pro	30	42	28
tRNA_Arg	49	74	95
tRNA_Trp	8	14	9
tRNA‐Sec	1	2	1
tRNA_Val	28	56	34
tRNA_His	12	26	9
tRNA_Thr	339	791	153
tRNA_Gly	26	64	80
tRNA_Met	17	51	28
tRNA_Asn	21	72	21
tRNA_Ala	76	465	93
Total	1985	2091	940
5_8S_rRNA	3	9	5
5S_rRNA	54	18	99
LSU_rRNA	6	36	9
SSU_rRNA	6	18	6
Total	69	81	119

### 3.3 Transposable elements in *O. edulis* and other oyster genomes

Repeated elements annotated in the five assemblies using the same pipeline method for proper comparison (Table 4) showed a similar content of various repeated elements, except for *P. martensii* (which is not a true oyster of Ostreidae like the other four species) for which TEs represent <15% of the genome. Amon the five species, *O. edulis* presents the highest coverage in TEs (38.05%). Although each element type is variably represented among species (e.g., Penelope elements are rare in both Crassostrea, as are SINEs in *C. gigas* and *P. martensii*), *O. edulis* almost always has the highest (LINEs, YR elements) or second‐highest (Penelope, SINEs, DNA transposons, rolling circles) proportions of TEs for each element type. Only LTR‐retrotransposons seem to be a little less frequent (1.61%) than in other species.

**TABLE 4:** Copy number and genomic content of the repetitive elements in *Ostrea edulis*, *Crassostrea gigas*, *Crassostrea virginica*, *Pinctada martensii*, and *Saccostrea glomerata* genomes generated following the annotation pipeline described in this paper.

	*Ostrea edulis*			*Crassostrea gigas*			*Crassostrea virginica*			*Pinctada martensii*			*Saccostrea glomerata*		
	% genome	Copies Nb	Nb of bp	% genome	Copies Nb	Nb of bp	% genome	Copies Nb	Nb of bp	% genome	Copies Nb	Nb of bp	% genome	Copies Nb	Nb of bp
Retroelements															
Penelope	4.46	57,601	38,234,354	0.49	4421	3,174,681	0.11	764	740,415	5.84	90,093	57,830,428	3.01	39,094	23,757,514
LINES	3.05	31,673	26,178,491	2.83	18,815	18,348,219	1.79	12,916	12,273,678	0.94	10,497	9,359,382	1.53	12,860	12,064,399
L2	0.43	4915	3,721,701	0.04	310	239,915	0.03	237	210,627	0.09	1076	925,824	0.28	1812	2,188,757
CR1	0.23	2999	2,005,708	0.37	2610	2,399,937	0.52	3921	3,564,568	0.20	2864	1,989,573	0.24	2135	1,900,378
RTE‐X	0.82	8206	7,016,657	0.82	6001	5,331,699	0.40	3138	2,769,765	0.18	2023	1,799,210	0.39	3578	3,071,346
I	0.33	5595	2,804,522	0.00	0	0	0.13	1491	883,115	0.00	0	0	0.07	867	565,958
L1	0.84	6494	7,179,616	0.75	3694	4,841,718	0.59	3301	4,023,806	0.29	2821	2,901,900	0.2	2489	2,529,348
RTE	0.33	2846	2,829,694	0.82	6001	5,331,699	0.07	461	456,806	0.15	1487	1,494,237	0.17	1548	1,331,519
Others	0.07	618	620,593	0.03	199	203,251	0.05	367	364,852	0.03	226	248,638	0.06	431	477,093
SINES	1.13	17,644	9,710,552	0.06	396	370,098	1.27	15,408	8,696,250	0.28	3787	2,755,480	0.65	11,734	5,149,632
LTR elements	1.61	9362	14,030,935	2.51	10,438	16,258,283	1.63	8440	11,191,940	1.00	6838	9,867,404	1.94	11,497	15,278,733
Gypsy	1.33	8012	11,588,206	2.07	9159	13,397,133	1.41	7519	9,650,060	0.88	6273	8,757,301	1.70	10,292	13,403,278
BEL/PAO	0.26	1237	2,229,921	0.42	1231	2,734,597	0.21	795	1,410,415	0.07	503	706,175	0.22	1144	1,745,192
Copia	0.02	79	189,857	0.02	48	126,553	0.01	45	100,528	0.00	0	0	0.02	61	130,263
Others	0.00	34	22,951	0.00	0	0	0.00	81	30,987	0.04	62	403,928	0.00	0	0
YR elements	0.65	5111	5,562,901	0.51	2588	3,317,411	0.33	1613	2,226,523	0.12	1028	1,163,186	0.37	2657	2,887,324
Ngaro	0.44	3150	3,810,113	0.34	1768	2,230,023	0.19	982	1,287,955	0.02	196	212,583	0.27	1810	2,115,549
DIRS	0.20	1961	1,752,788	0.16	820	1,087,388	0.14	631	938,568	0.10	832	950,603	0.10	847	771,775
DNA transposons	7.08	83,982	60,748,905	7.32	74,071	47,420,818	5.34	46,434	36,532,449	3.56	57,625	35,264,480	5.88	69,471	46,338,311
Maverick	0.28	751	2,438,022	0.22	764	1,393,542	0.38	1133	2,580,104	0.19	991	1,897,408	0.08	701	600,613
TcMar/Pogo	1.38	14,389	11,848,554	1.19	12,709	7,710,105	0.49	4683	3,333,461	0.48	7725	4,471,920	1.38	13,911	10,870,057
Hobo/Ac/Tam	0.95	12,619	8,173,678	0.39	3002	2,520,162	0.46	4165	3,166,922	0.80	12,909	7,915,023	0.49	5663	3,866,272
Zator	0.38	4849	3,295,890	0.15	1481	960,144	0.04	425	293,547	0.15	3014	1487 162	0.14	1896	1,064,820
Crypton	1.37	20,899	11,730,201	1.78	22,194	11,503,648	1.09	12,912	7,489,797	0.75	15,571	7,448,289	1.32	19,279	10,442,231
Academ	0.25	2649	2,153,984	0.03	167	213,307	0.13	651	894,907	0.08	791	743,310	0.03	229	274,905
EnSpm	0.09	705	809,979	0.32	1788	2,080,891	0.47	3155	3,220,923	0.16	2902	1,579,436	0.31	2877	2,450,110
MULE/MuDR/IS905	0.13	1341	1,130,079	0.04	328	288,206	0.10	806	672,632	0.07	875	678,893	0.08	701	600,613
PHIS	1.22	12,327	10,474,972	1.56	14,069	10,077,041	1.14	9623	7,838,419	0.66	10,261	6,499,076	1.09	13,198	8,619,218
Kolobok	0.58	8162	5,001,169	1.24	14,339	8,041,774	0.61	6107	4,177,778	0.03	385	334,047	0.65	7877	5,160,850
PiggyBac	0.11	1710	965,878	0.15	923	962,318	0.13	485	901,089	0.00	0	0	0.09	1064	748,137
Sola	0.16	1741	1,401,559	0.15	896	988,546	0.13	855	871,136	0.11	990	1,119,636	0.09	673	703,386
Others	0.15	1840	1,324,940	0.11	791	681,134	0.16	1434	1,091,734	0.08	1031	820,280	0.12	1402	937,099
Rolling circles	9.88	110,094	84,753,110	12.47	79,436	80,781,395	9.48	72,591	64,896,885	3.83	54,233	37,948,915	9.88	110,094	84,753,110
Unclassified	10.20	131,922	87,508,527	3.90	31,005	25,285,376	8.74	82,096	59,864,032	0.09	1296	867,014	11.24	131,498	88,620,649

**FIGURE 2:**
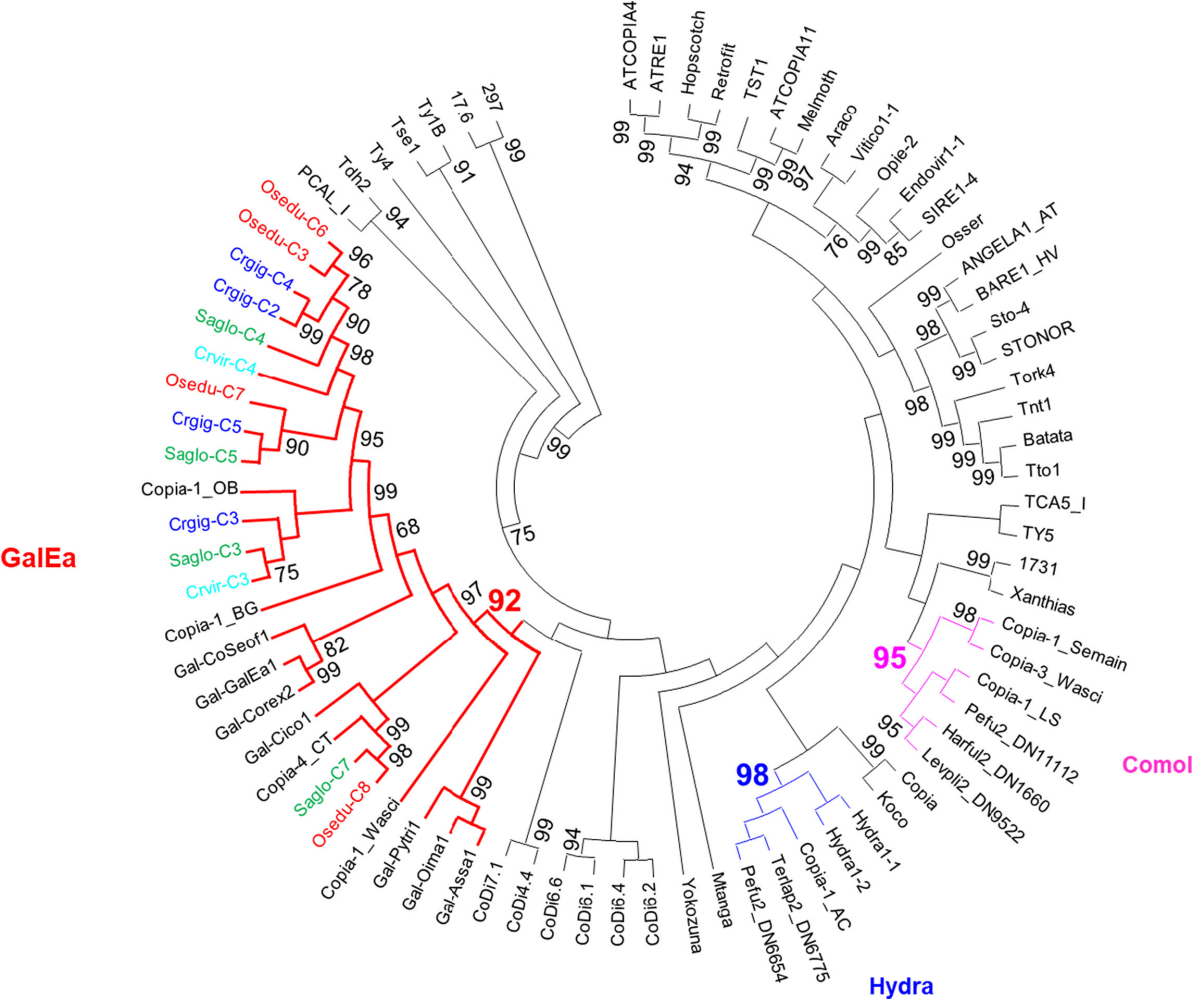
Phylogenetic relationships of Copia retrotransposons. The tree is based on neighbor‐joining analysis of RT/RNaseH domain amino acid sequences. The Copia subfamilies from oysters are indicated in color: *Crassostrea gigas* in dark blue, *Crassostrea virginica* in light blue, *Ostrea edulis* in red, *Pinctada martensii* in purple, and *Saccostrea glomerata* in green. Node statistical support values (>70%) come from nonparametric bootstrapping using 100 replicates.

In order to characterize LTR‐retrotransposons in oyster genomes, LTRharvest was used and its output was integrated into phylogenetic analyses. For this purpose, two types of sequences were used: (i) either a consensus devoid of possible insertions in the case of a cluster of elements previously defined by Uclust or (ii) or isolated (single) sequences that did not cluster with any other elements. A total of 797 subfamilies were detected by LTRharvest in the five genomes, including 14 Copia, 155 BEL/Pao, and 628 Gypsy (Table S2). The relative abundance of the three superfamilies in *O. edulis* (76% for Gypsy elements, 22% for BEL/Pao elements, and 2% for Copia elements) is quite similar among oyster species; except for Copia elements in *P. martensii* only represented by one single copy (Table 5). The phylogenetic tree of Copia elements revealed that all elements belong to the GalEa clades (Figure 2).

**TABLE 5:** Genomic proportions of the different clades of LTR‐retrotransposons detected in *Ostrea edulis*, *Crassostrea gigas*, *Crassostrea virginica*, *Pinctada martensii*, and *Saccostrea glomerata* genomes.

	*O. edulis*	*C. gigas*	*C. virginica*	*P. martensii*	*S. glomerata*
Genome size	872,374,424 bp	647,887,097 bp	684,723,884 bp	99,0984,031 bp	788,100,799 bp
GalEa	0.022 (4)	0.020 (4)	0.015 (2)	s	0.017 (4)
Flow	s	0.015 (1)	–	0.003 (1)	s
Suzu	0.005 (1)	0.015 (2)	0.006 (1)	s	0.006 (1)
Tas	0.027 (4)	0.019 (2)	0.041 (3)	–	–
Sinbad	0.034 (5)	0.036 (3)	0.027 (2)	0.004 (2)	0.015 (2)
Sparrow	0.187 (26)	0.300 (33)	0.127 (16)	0.055 (9)	0.161 (25)
	s	0.021 (2)	0.005 (1)	0.004 (1)	0.011 (2)
Unclassified	0.002 (1)	0.017 (2)	–	0.005 (1)	0.029 (6)
Pao/BEL	0.256	0.422	0.206	0.071	0.221
AB‐clade	0.034 (6)	0.039 (7)	0.025 (3)	–	0.034 (8)
C‐clade	0.142 (28)	0.117 (13)	0.138 (18)	0.053 (16)	0.133 (23)
MolGy1	0.347 (23)	0.376 (32)	0.290 (25)	0.075 (6)	0.319 (26)
MolGy2	0.584 (47)	0.631 (51)	0.450 (29)	0.208 (21)	0.672 (52)
MolGy3	0.062 (11)	0.039 (5)	0.039 (6)	0.024 (6)	0.053 (10)
MolGy4	0.014 (1)	0.250 (7)	s	0.213 (4)	0.044 (2)
MolGy5	0.050 (2)	0.005 (1)	0.099 (2)	0.220 (8)	0.121 (3)
MolGy6	–	0.216 (11)	0.079 (6)	0.018 (2)	0.070 (4)
MolGy10	0.006 (1)	0.018 (1)	0.011 (1)	–	0.013 (2)
MolGy17	0.0182 (2)	s	s	0.007 (1)	0.011 (2)
MolGy18	0.005 (1)	0.006 (1)	–	0.003 (1)	0.010 (1)
MolAn	0.026 (2)	0.262 (16)	0.223 (10)	s	0.132 (14)
Unclassified	0.041 (7)	0.109 (8)	0.056 (9)	0.064 (12)	0.089 (11)
Gypsy	1328	2068	1409	0.884	1701
LTR‐retrotransposons	1606	2509	1630	0.955	1939

Note

The number of subfamilies of each clade is given in brackets. “Unclassified” = elements not linked to a clade; “−” = no element detected; “s” = only single sequence detected.

The *O. edulis* genome did not contain elements of the BEL, Pao, and Dan clades (Figure S3) but contained TEs in the six other clades in the BEL/Pao superfamily. Most of its elements belong to the Sailor lineage, mostly to the Sparrow, Sinbad, and Tas clades and only one subfamily in the Suzu clade and one element in the Flow clade are present. This distribution is globally observed in the other oysters, with a large Sparrow clade. Only *O. edulis* and *C. gigas* present all the six clades, in particular, the absence of the element of the Tas clade in both *P. martensii* and *S. glomerata*.

**FIGURE 3:**
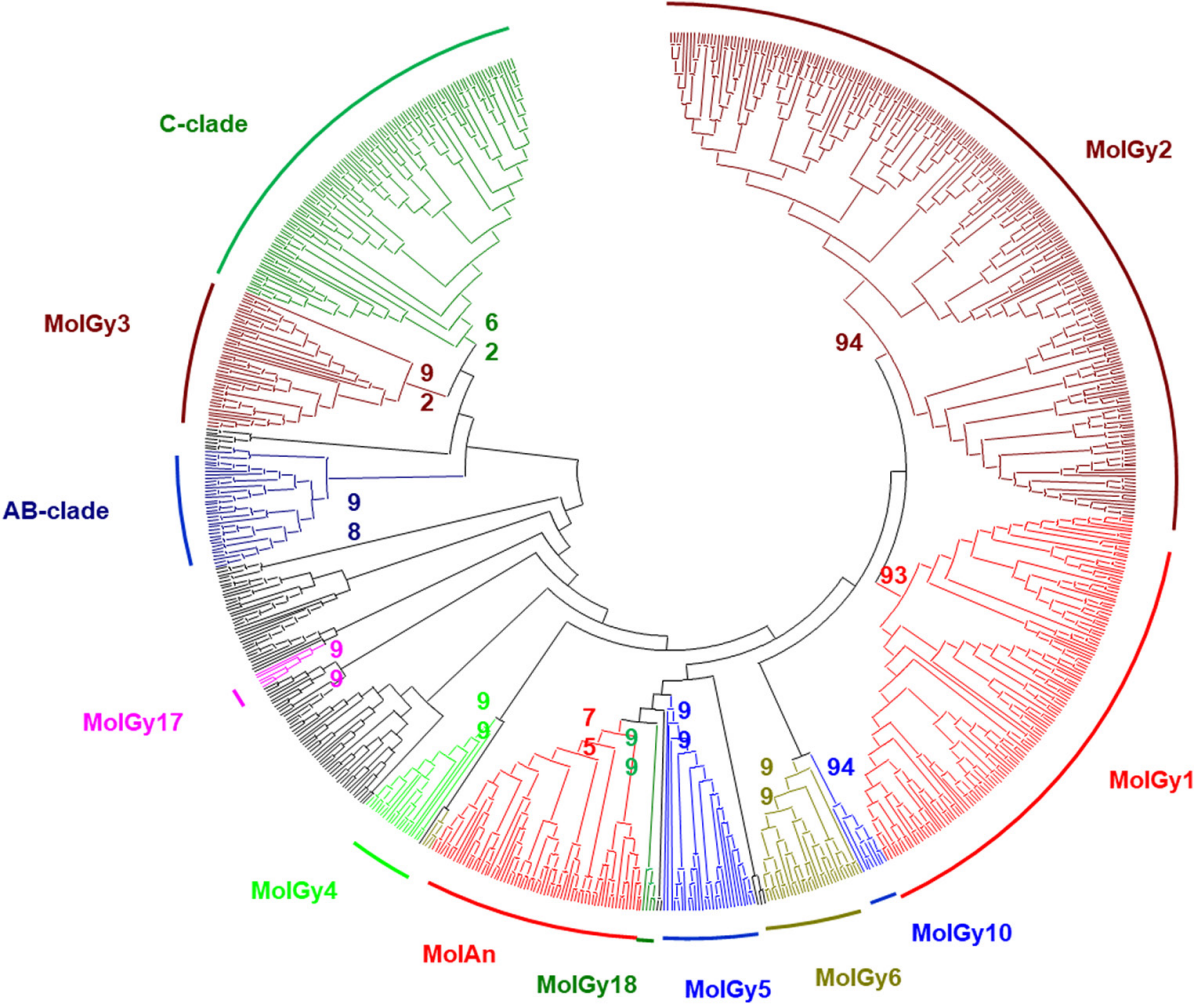
Phylogenetic relationships of Gypsy retrotransposons. The tree is based on neighbor‐joining analysis of RT/RNaseH domain amino acid sequences. The clades that have elements of oysters are indicated in color, and the clades not present in oysters are represented by the black lines. Node statistical support values (>70%) come from nonparametric bootstrapping using 100 replicates.

Gypsy superfamily tree reveals 12 clades in oysters (Figure 3, Figure S4). Ten correspond to the MolGy clades previously defined from mollusc elements (except for clades MolGy11 and MolGy13) and to the clades A‐B and C, also known to have Gypsy elements of molluscs. Two new putative clades can be further identified with oyster elements: MolGy17, observed in all five species, and MolGy18 that is only absent in *C. virginica*. *O. edulis* does not present any element of the clade MolGy6, although it is quite well represented in the four other species; and it also has very few elements of the MolAn clade (Table 5). Three clades (Molgy1, Molgy2, and the C‐clade) are well recovered, represent about 1.26% of the genome, and largely dominate with three‐quarters of the subfamilies as observed in other oysters.

### 3.4 Differential expression analysis in *M. refringens‐*infected oysters

**FIGURE 4:**
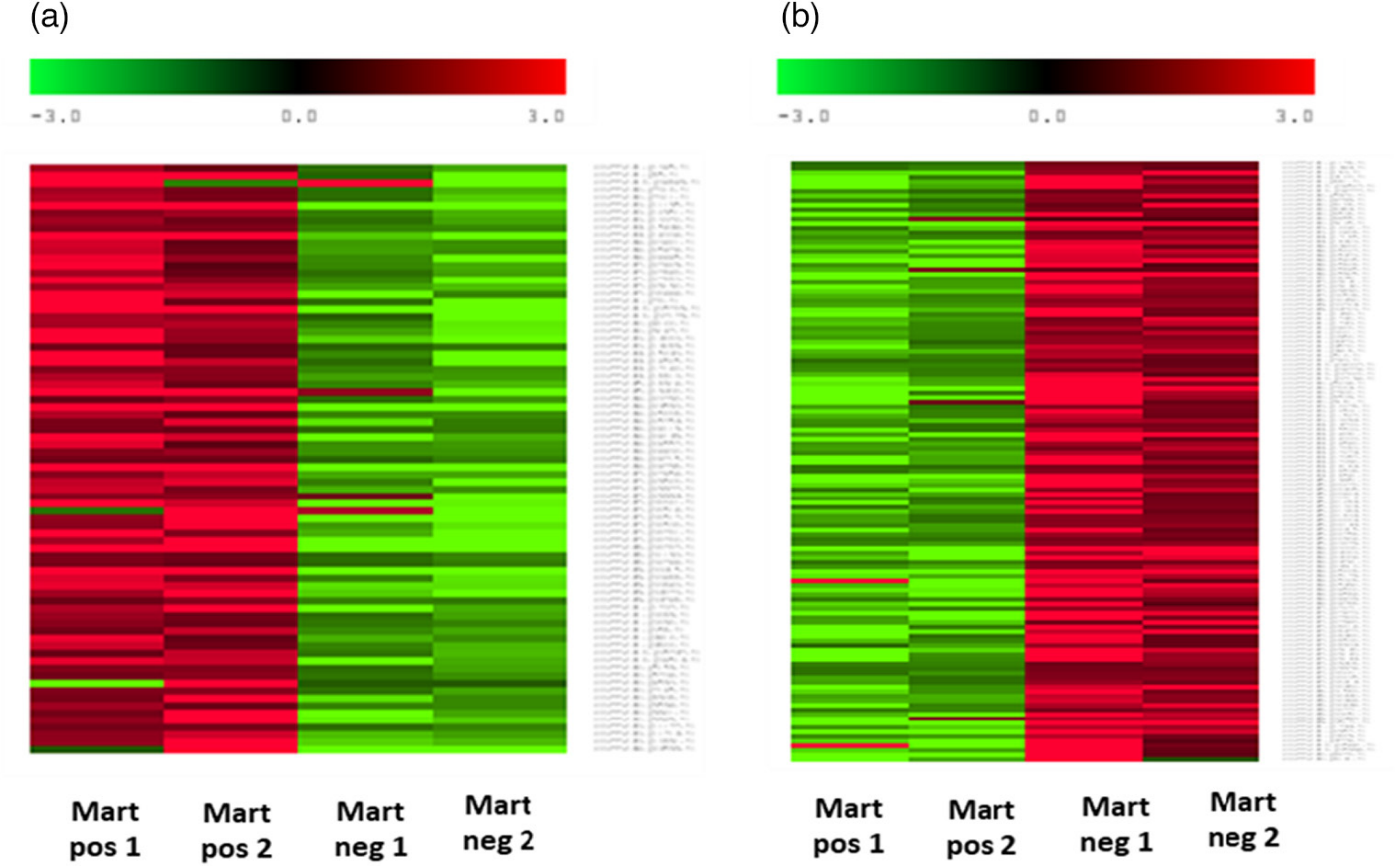
Heat maps of the most upregulated DEGs (a) and the most downregulated DEGs (b) in hemocytes of *M. refringens*‐infected *O. edulis* individuals. The heat map shows the matrix of fold changes that were calculated for each condition and each DEG by normalizing the expression of each condition and DEG to the expression of the DEG in all conditions. Positive fold changes are in red, and negative fold changes are in green.

In the hemocytes, seventy‐eight DEGs are upregulated (Figure 4a) where 4 terms in the Biological Process category (GO:0006259 DNA metabolic process, GO:0090305 nucleic acid phosphodiester bond hydrolysis, GO:0006302 double‐strand break repair, GO:0007155 cell adhesion), and 2 terms in the Molecular Function category (GO:0003682 Chromatin Binding and GO:0003677 DNA binding) are enriched (Table S4). Among the most downregulated regulated genes, we identified IAPs, mannose receptor C‐type, serine protease inhibitor, one SOD, and several genes encoding C1q complements, which are all involved in the immune response. Among upregulated genes, we identified several proteins involved in apoptosis such as ced‐1 and other genes involved in cell adhesion such as a sushi, von Willebrand factor type A, an EGF, and the pentraxin domain‐containing protein 1 and several TRIM proteins. A total of 143 DEGs are downregulated in *M. refringens*‐infected oysters (Figure 4b). GOterm enrichment analysis shows that 4 terms in the Biological Process category (GO:0007155 cell adhesion, GO:0032715 negative regulation of interleukin‐6 production, GO:0032088 negative regulation of NF‐kappaB transcription factor, and GO:0070373 negative regulation of ERK1 and ERK2 cascade), 5 terms in the Cellular Component category (GO:0005581 collagen trimer, GO:0005576 extracellular region, GO:0005615 extracellular space, GO:0031012 extracellular matrix, and GO:0030054 cell junction) and 3 terms in the Molecular Function category (GO:0005102 receptor binding, GO:0030020 extracellular matrix structural constituent conferring tensile strength and GO:0005518 collagen binding) are significantly over‐represented in the downregulated DEGs (Table S3).

**FIGURE 5:**
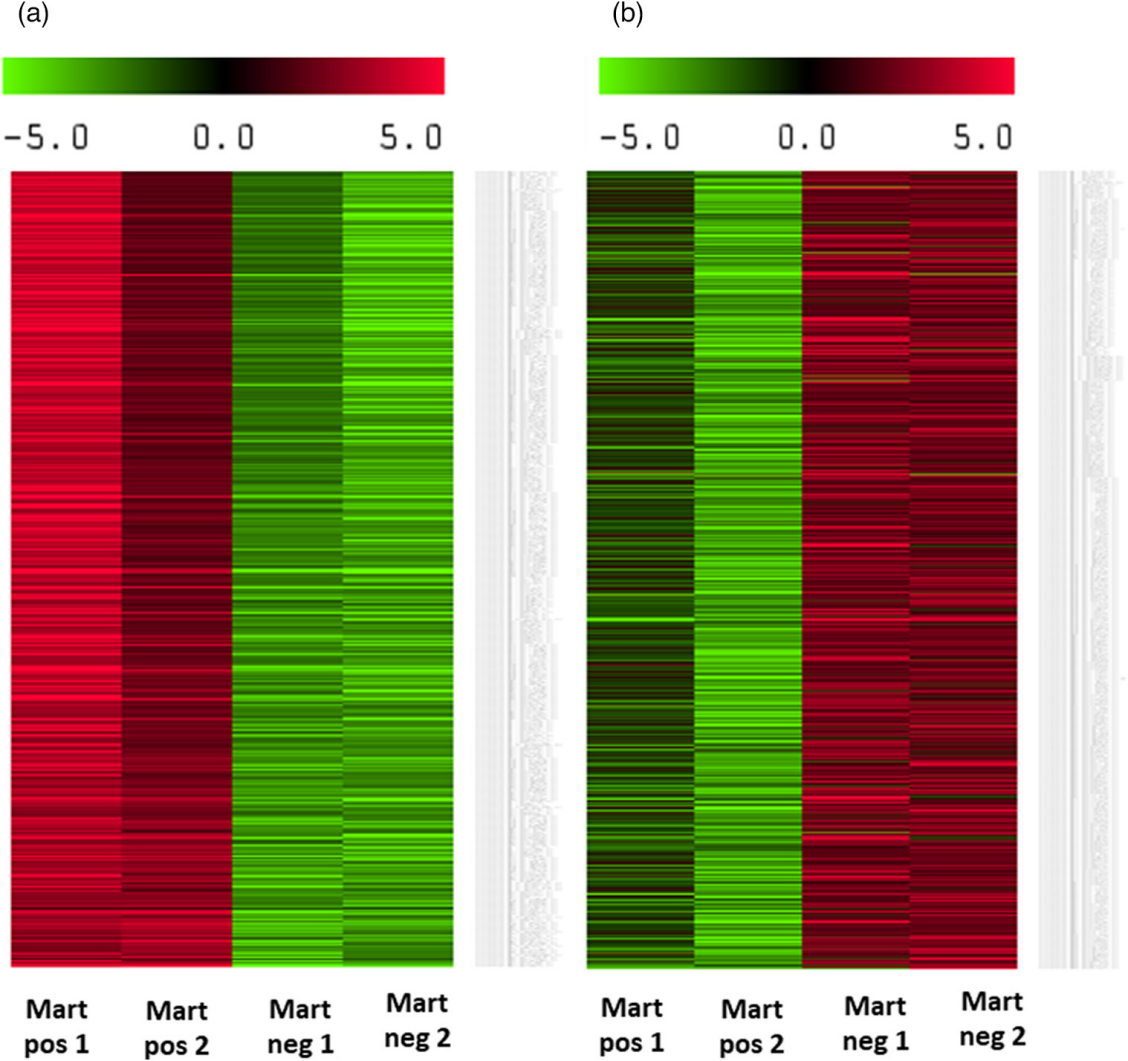
Heat maps of the most upregulated DEGs (a) and the most downregulated DEGs (b) in the digestive gland of *M. refringens*‐infected *O. edulis* individuals. The heat map shows the matrix of fold changes that were calculated for each condition and each DEG by normalizing the expression of each condition and DEG to the expression of the DEG in all conditions. Positive fold changes are in red, and negative fold changes are in green.

In the digestive gland, a total of 926 DEGs have been identified as over‐expressed in *M. refringens*‐infected oysters (Figure 5a) and represent 29 terms in the Biological Process category, 28 terms in the Cellular Component category, and 12 terms in the Molecular Function category (Table S5). Among the genes showing the strongest upregulations in *M. refringens*‐infected individuals (log2 fold >4), several genes are known to be involved in immune response processes or cancer‐like pathologies (prominin, DMBT1, 2‐proprotein convertase subtilisin/kexin), cell adhesion (contactin, laminin). Several genes involved in DNA, RNA, or histone methylation process are also upregulated (log2 fold >2.5) such as lysine methyltransferase. We also identified a set of proteins known to specifically interact with microorganisms such as meprin (three genes), coiled‐coil domain‐containing proteins (18 genes), complement component 1q (11 genes), and GTPase, IMAP family member proteins (seven genes), and four genes encoding the interferon‐induced protein. Specific genes involved in the late response to apoptosis such as caspase 3 and 6 are also identified. We also identified members of transposable elements (3 Pif transposases, 3 LINE‐1 transposase, and 3 Tigger transposable element).

We identified 1640 DEGs downregulated in *M. refringens*‐infected oysters (Figure 5b) representing 68 terms in the Biological Process category, 22 terms in the Cellular Component category, and 33 terms in the Molecular Function category (Table S6). Among those DEGs, we identified three genes encoding Cadherin 12 that are involved in cell adhesion homeostasis, one gene involved in the protection of germline (Mage protein), one gene encoding the hemocytin, which is involved in hemocyte aggregation, and a gene encoding ADAR that is involved in RNA editing. Several genes involved in the positive regulation of transcription from RNA polymerase II promoter and 14 genes encoding G‐protein‐coupled receptors that convert extracellular signals into intracellular responses have been identified. We also detected a total of 77 genes encoding several families of solute carrier proteins (SLCs) as being downregulated in the presence of *M. refringens*. A set of genes also known to be involved in relationships with microorganisms have been detected including ABC transporters (10 genes), ADAM proteins (six genes), several CYP proteins (five genes), several proteins containing a tyrosine phosphatase or kinase activity (35 genes), 22 members of the transmembrane proteins family (TMEM), nine genes containing a von Willebrand factor D and EGF domains (VWDE), several LDL receptor‐related proteins (11 Lrp genes), and six genes encoding carboxypeptidases. Four genes belonging to the B‐cell lymphoma two family that are involved in the apoptotic response, including two proapoptotic members (Bad, Bak) and two antiapoptotic members (BCL2), have been evidenced.

### 3.5 SLCs distribution in *O. edulis* genome

**FIGURE 6:**
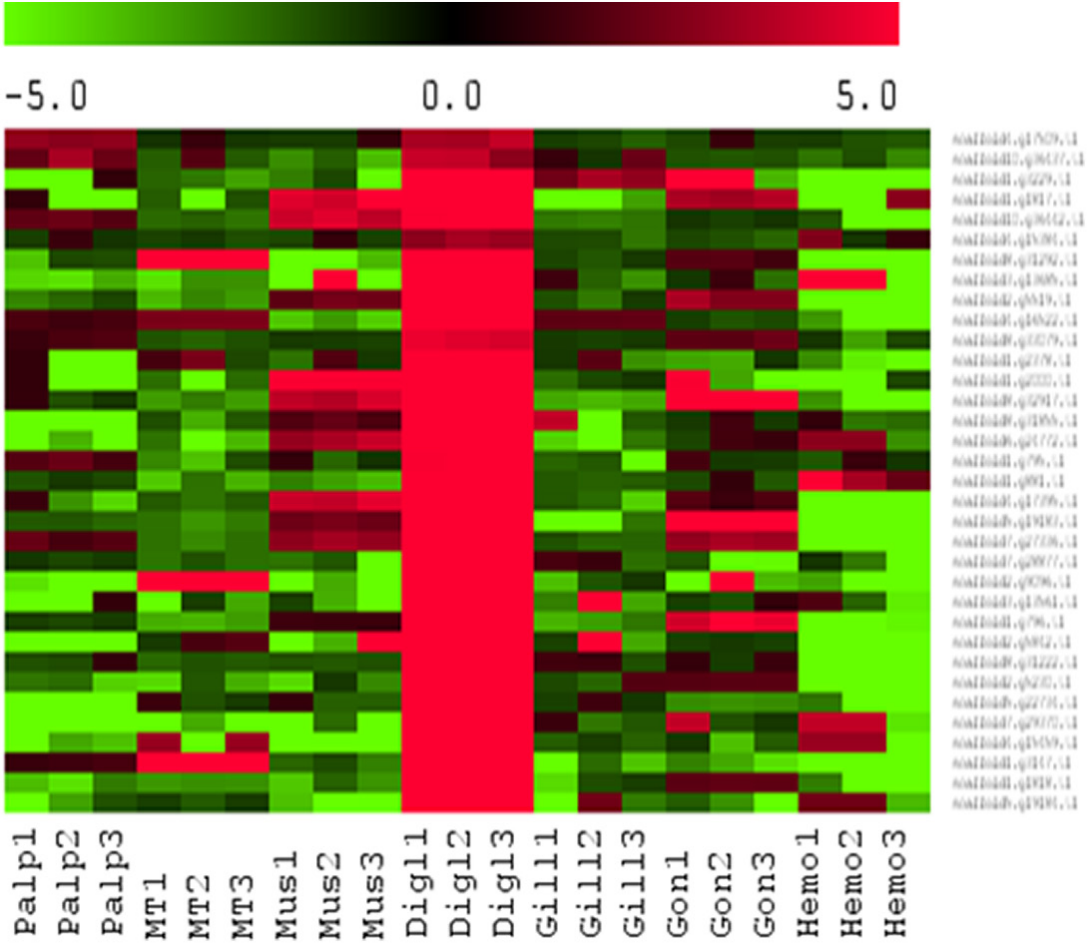
Heat map of the 30 most upregulated SLCs in the digestive gland (Digl) of *Ostrea edulis* compared with palp (Palp), mantle (MT), adductor muscle (Mus), gills (Gill), gonads (Gon), and hemocytes (Hemo). The heat map shows the matrix of fold changes that were calculated for each tissue by normalizing the expression of each SLC within a tissue to the expression of the SLC in all tissues. Positive fold changes are in red, and negative fold changes are in green.

A total of 511 SLC genes classified in 48 families (*e*‐value < 10^−4^) were identified, which is similar to what was observed in *C. gigas* (510 SLCs) but lower than the 673 genes identified in the scallop *P. yessoensis* (Xun et al., 2020). The distribution of SLC across the 10 chromosomes showed differences in SLC repartition with a lower number of SLC genes on chromosomes 6, 9, and 10 (Table S7). The number of SLC genes per family strongly varies and SLC5, SLC6, SLC16, SLC17, SLC25, and SLC39 families have more than 20 genes. An enrichment in SLC16 (70 genes) is detected in *O. edulis* (versus 54 in *C. gigas* and 75 in *P. yessoensis*) and 28 of the copies are present in the chromosome 8 and 11 in the chromosome 1 including a cluster of 8 genes. The SLC6 family is mainly present in the chromosome 1 (24 copies) and 2 (16 copies) and the SLC5 is mainly present in the chromosome 5. We analyzed the expression of the 511 SLC in the different organs used for the genome annotation (Table S7) and showed that 91 SLCs are significantly upregulated in the digestive gland compared with all other organs (Figure 6). Among these 91 genes, 26 are among the downregulated SLCs identified in the *M. refringens*‐infected digestive gland. The largest number of digestive gland over‐expressed SLCs are from SLC16 (15), SLC6 (16), PySLC5 (9), and SLC22 (6).

## 4 DISCUSSION

The use of the combined PacBio long reads, Illumina shotgun, and 10X sequencing approaches allowed us to obtain a high‐quality reference genome assembled at a chromosomal level for the flat oyster *O. edulis*, which was used for subsequent genomic analysis. With a size of 887 Mb distributed along the 10 predicted chromosomes and good assembly statistics, the *Roscoff_O.edulis‐V1* assembly ranks well among other sequenced genomes of bivalve molluscs (Farhat et al., 2022; Li et al., 2021; Peñaloza et al., 2021). The *Roscoff_O.edulis‐V1* genome size is larger than that of the Crassostreidae (around 600 Mb), but the number of protein‐coding genes predicted (35,962) is close to that obtained in other oyster species such as *C. gigas* (31,371), *C. virginica* (34,587), and *P. fucata* (31,477).

### 4.1 Analysis of tRNA and miRNA reveals specificity in *Ostrea* genome

Transfer RNAs play an essential role in cellular life and in many metabolic processes (Francklyn & Minajigi, 2010). In many species, the number of genes encoding each tRNA generally correlates with the amino acid frequency with some exceptions such as in *Arabidopsis thaliana*, where a higher number of genes encoding tRNAs‐Tyr, Ser, and Pro are detected (Michaud et al., 2011). Counting of serine residues in the *O. edulis* proteome does not reveal an excess of this residue over other amino acids, failing to explain the high tRNA‐Ser copy number. The frequency of serine amino acid in the proteomes of three other molluscs (*C. gigas*, *C. virginica*, and *M. mercenaria*) does not show any significant difference (ranging from 7.9% to 8.28%). The same result was obtained when considering the proportion of threonine residues in the proteome despite a high number of tRNA‐Thr genes in *O. edulis*. The number of tRNA genes identified in the three species we analyzed is consistent with what is found in other molluscan species such as *Scapharca broughtonii* with 1541 tRNA genes (Bai et al., 2019) or *C. gigas* with 641 tRNA genes (Peñaloza et al., 2021). In eukaryotes, no general trend in the number of tRNA genes is evident and the strong variations observed could reflect differences in the evolutionary history of lineages. While the study of tRNA genomic organization and structural evolution remains partial (Goodenbour & Pan, 2006), tRNA genes have been shown to play a role in genome organization by acting as barriers to DNA replication fork progression (McFarlane & Whitehall, 2009) or by contributing to chromosomal instability (Admire et al., 2006). No clear relation between tDNA copy number and their organization in clusters in genomes is evident, and most tDNA clusters are small, containing only a few colocalized tRNA genes with the exception of Nematostella and some fish species (Bermudez‐Santana et al., 2010). Despite a high number of the tRNA‐Ser and tRNA‐Thr that also present a high disequilibrium in the proportion of isoacceptors in the *Roscoff_O.edulis‐V1* genome, no specific clustering of those tRNA genes is observed. Variations in codon usage might influence the variation in tDNA copy number (Rocha, 2004), but no strong disequilibrium for Ser resides is evident in *O. edulis* (Gerdol et al., 2015). Differences in the number of rDNA loci between individuals of the same species (e.g., *M. galloprovincialis*), and in the location of the rDNA loci between different cells from the same individual have been found (Insua & Mendez, 1998). Additional analyses are needed to better understand whether and how the enrichment observed for the tRNA‐Ser and tRNA‐Thr plays a functional role in *O. edulis* physiology because this specific pattern is not observed in other oyster genomes and no difference in amino acid composition has been detected between proteomes.

Even though molluscs represent the second largest phylum in Metazoa, only a limited number of studies have been performed on the identification and the role of miRNA despite their essential role in cellular development, proliferation, apoptosis, oncogenesis, differentiation, and disease (Bueno & Malumbres, 2011). While more than 48,800 miRNA are available in all species (www.mirbase.org), only 245 families have been identified in molluscs mainly due to the low number of genomes available, a lack of specific miRNA annotation, and a limited number of miRNA transcriptomic analysis. The comparison of the miRNAs copy number shows differences within a species between the different approaches used for their annotation limiting the ability to make reliable comparisons. In *C. gigas*, eight copies of miRNA‐184 are identified by Huang et al. (2021) versus 44 by Rosani et al. (2021). Further, differences are observed when comparing predicted miRNA and miRNA identified in small‐RNAseq sequencing, which do not confirm that the predicted miRNAs are actually expressed. In the *Roscoff_O.edulis‐V1*, we identified several miRNA families that exhibit high copy numbers distributed along the 10 chromosomes without specific clustering. The most represented family is miRNA‐148 with 219 copies, which is known to act as a negative regulator of MyD88‐dependent NF‐κB signaling in the teleost fish (Chu et al., 2017) and inhibited in the herbivorous carp *Ctenopharyngodon idella*, in response to bacterial infection (Fang et al., 2020). The Mir‐841 gene with 106 copies in *O. edulis* genome is involved in response to metabolic stress (Nischal et al., 2014) or virus (Moyo et al., 2017) in the plants. The involvement of several miRNAs in response to the herpes virus OsHV‐1 has been demonstrated in *C. gigas* (Rosani et al., 2020), *Chlamys farreri* (Chen et al., 2014). In *O. edulis*, miRNA involved in immune system regulation (miRNA1, miRNA10, miRNA8, miRNA33) has been shown to be regulated in response to *B. ostreae* (Martín‐Gómez et al., 2014). Further work should focus on coupling miRNA prediction and miRNA transcriptomic profiling in *O. edulis*.

### 4.2 Transposable elements

A comparison of the TE composition in the four Ostreidae species and the Pteriidae species analyzed shows a relative homogeneity of their content. The percentages of genome coverage are close for most types of elements (LINEs, SINEs, DNA transposons, YR elements) and the only remarkable difference in *O. edulis* is the over‐representation of Penelope elements (4.46% vs. 0.5% in other species), and a global under‐representation of LTR‐retrotransposons (1.61% in *O. edulis* vs. 4.37%). With the exception of the MolGy6 clade, *O. edulis* possesses all the clades detected in oysters, and 17 of the 23 clades described in molluscs are present in oysters. A targeted study of Gypsy elements within 20 bivalve genomes revealed 20 branches within the Gypsy clade C (Farhat et al., 2022), and 18 are found in oyster genomes. Analysis of the five genomes has further defined two new clades, MolGy17 and MolGy 18 that appear to be specific to Ostreidae and Pteriidae. Specific elements such as the Steamer LTR‐retrotransposon family, which is associated with neoplasia in other bivalve species (Metzger et al., 2018) have not been detected in *O. edulis* genome while it is present in other oysters like *C. gigas*, *C. virginica*, and *S. glomerata* (Farhat et al., 2022). Contrary to *C. gigas*, no specific enrichment in the rolling‐circle transposable elements Helitrons is detected in *O. edulis*. However, the high percentage of unclassified TEs in both *O. edulis* and *S. glomerate* genomes suggests that flat oysters could contain new families of ETs not yet described. Transposable elements are known to have a large impact on genome structure and stability, and are therefore considered to play an important role in many evolutionary and ecological processes such as biodiversity generation, stress response, adaptation, speciation, and colonization mechanisms in eukaryotes (Biemont & Vieira, 2006; Finnegan, 2012). Environmental variations can promote genome plasticity through transcriptional activation and TEs mobilization, often in response to specific stimuli such as biotic stresses (pathogen) or abiotic environmental changes (temperature, heavy metals, UV) (Capy et al., 2000; Melayah et al., 2001). This variability transmitted to the offspring would also favor a better adaptation of the organisms to environmental changes (long‐term effect detectable in the genome). Since several TEs appear to be regulated in response to *M. refringens*, an extensive analysis of TE regulation in response to biotic and abiotic environmental parameters should be conducted to better understand the role of TE in flat oyster biology.

### 4.3 Transcriptomic regulation in response to *M. refringens*

Studies of the responses of *Ostrea edulis* to the presence of *M. refringens* have not yet been carried out, despite the strong impact of *M. refringens* in terms of mortality on the oyster beds. The present study carried out on individuals naturally infected by *M. refringens*, although preliminary, highlighted some metabolic pathways classically involved in parasite interaction but also new pathways that could be more specifically associated with the response to a eukaryote parasite. The low number of genes regulated in hemocytes in response to *M. refringens* indicates that despite its key role in immunity, this tissue appears to play only a minor role in the response to this parasite. On the contrary, the high number of differentially regulated genes and corresponding biological functions in the digestive gland emphasizes the systemic response of *O. edulis* to *M. refringens*. In hemocytes, a downregulation occurred for genes encoding IAPs, mannose receptor C‐type, serine protease inhibitor, and C1q complement that all contribute to the molecular response to parasites (Chan et al., 2021; Li et al., 2019). In the digestive gland, 11 genes coding for C1q different from those identified in hemocytes are instead upregulated demonstrating differential regulation of this protein family. C1q domain‐containing proteins are characterized by a large diversity of gene number across species (Farhat et al., 2022; Gerdol et al., 2019; Mun et al., 2017; Peng et al., 2020) and have been shown to be over‐represented in *O. edulis* genome (Gundappa et al., 2022). Further studies will therefore be necessary to better understand the recognition specificities of this set of molecules. Serine protease inhibitor is involved in *Perkinsus marinus* resistance in oyster *C. virginica* (He et al., 2012; Yu et al., 2011) and their downregulation in *M. refringens*‐infected oysters may reflect a lower ability to inhibit the serine proteases produced by the parasite. In both tissues, the increased expression of genes involved in apoptosis is coherent with previous results obtained on *O. edulis* in response to *B. ostreae* (Gervais et al., 2019) or *C. virginica* in response to *Perkinsus marinus* (Lau et al., 2018) In the digestive gland, several effector caspases (casp 3 and casp 6) are over‐expressed in infected individuals while paradoxically, two pro‐apoptotic genes involved in the early phases of regulation of this process (Bak and Bad genes) and two antiapoptotic genes (BCL2) are inhibited, reflecting the existence of control on the pro‐anti apoptosis balance. The apoptotic response in molluscs that involves many molecular actors (Kiss, 2010; Witkop et al., 2022) is under the control of a wide diversity of molecular modulators and understanding their regulation by *M. refringens* remains an area of research to be explored further.

The identification of genes previously shown to respond to other models of parasite infection confirms that the transcriptomic response observed may result from the *M. refringens* effects, highlighting the complexity of the molecular targets affected/modulated by this parasite. We particularly found that genes involved in cilium motility or assembly or genes encoding G‐protein‐coupled receptors (14 genes) that are involved in the regulation of several cellular processes by sensing molecular cues outside the cells may play a role in the immune response (Bezares‐Calderon et al., 2020; He et al., 2015; Song et al., 2015). The identification of increased expression in infected oysters of several genes involved in DNA, RNA, or histone methylation process suggests that epigenetic processes could also participate in the response to *M. refringens*. Indeed, a growing number of studies suggested that protozoan parasites such as *Leishmania*, *Toxoplasma*, and *Theileria* manipulate host cells via epigenetic modification of host gene expression mainly resulting in permanent downregulation of host defense mechanisms to promote intracellular replication and survival of the pathogen (McMaster et al., 2016). As the number of studies on the regulation of methylation processes by parasites in molluscs is limited, the case of the *M. refringens*‐*O. edulis* interaction could pave the way for a new research axis. In response to *M. refringens* infection, the downregulation of a large set of genes encoding SLC is detected. The immune system coordinates complex signals to support the proliferation, differentiation, and effector function to protect cells against tumors and infections and SLCs play an important role in these regulations and participate in the modulation of various metabolic pathways by import and export of a large variety of small molecules (nutrients, drugs…) across biological membranes (Rives et al., 2017). Specific members of SLC families (SLC16A4, SLC39) play a role in viral replication resistance factor, providing a potential protective effect against the disease (Fisel et al., 2018; Liao et al., 2016) and members of both families are regulated in response to *M. refringens*. Changes in SLC expression are consistent with a disruption of many processes that depend on it and would explain the cascade of associated regulations. Strong alterations of these processes could conduct to the decrease in the condition index, loss of glycogen, discoloration of the digestive gland, growth arrest, tissue necrosis, and finally death of *M. refringens*‐infected oysters. SLC genes have also been shown to be under directional selection (Li et al., 2021), suggesting their role in adaptative response to environmental parameters (Kenkel et al., 2013; Martinez Barrio et al., 2016; Zhou et al., 2018). *M. refringens* seems to act by inhibiting certain processes normally associated with an activation of the immune response. This is the case of the repression of the ADAM proteins which belong to the matrix‐metalloproteinases family. They allow the detachment of adhesion proteins which play a central role in attracting inflammatory cells to sites of tissue damage and regulate the activation and phagocytic activity of immune cells (Rahn & Becker‐Pauly, 2022). Some ADAM genes might have a protective role against some parasites (Geurts et al., 2012). We observed an upregulation of several GTPase IMAP family members in response to *M. refringens*. Those genes have been shown to be upregulated in *Oncorhynchus mykiss* in response to the myxozoan *Ceratonova shasta* (Barrett & Bartholomew, 2021) or in the mice in response to *Toxoplasma gondii* infection (Kim et al., 2018). If the way these genes might mediate resistance is unclear, one possible mechanism may be through mediating the effects of interferons (IFN pathway), which orchestrates many cellular pathways and regulates the expression of hundreds of genes. In *O. edulis*, associated with the over‐expression of IMAP genes, we observed the over‐expression of several interferons induced proteins which are known to act as a primary way of defending against some parasite infections (Baerwald, 2013). In the same order, many proteins exhibiting a coiled‐coil domain are upregulated in response to *M. refringens* suggesting the presence of a strong cytoskeletal remodeling related to the presence of the parasite in the tissue. The coiled‐coil domain‐containing (CCDC) proteins have been implicated in many physiological processes including interactions with molecular components of signaling pathways and cytoskeletal polymerization, and they are implicated in the pathogenesis of a large number of cancers (Priyanka & Yenugu, 2021). Some parasites appear to use the low‐density lipoprotein receptor (LDLR) pathway of their host to facilitate their development and invasion of host tissue (Nagajyothi et al., 2011). The low‐density lipoprotein receptor (LDLR) family is composed of a class of transmembrane glycoproteins that bind and internalize extracellular ligands for degradation by lysosomes. Their downregulation in infected oysters may reflect an alteration in the functioning of lysosomes, limiting their intervention in the digestion of parasites that could be endocytosed before their degradation. Interestingly, we also observed a downregulation of seven genes encoding proteins associated with lysosomal membrane or enzymes such as acid phosphatases. A large set of transmembrane proteins (TMEM) are downregulated in response to *M. refringens*. TMEM acts as channels to allow the transport of specific substances across the biological membranes. Their specific functions remain poorly described, but some of them are involved in modulating cancer cell dissemination and metastasis formation (Marx et al., 2020; Qiao et al., 2016). No data on their role in response to parasites are available in molluscs but with 170 putative genes detected in *O. edulis* genome, the understanding of their role in response to parasites will require additional studies. Complementary analyses involving a larger number of individuals could be undertaken to better understand the entire process of response to this parasite and to identify putative genetic loci associated with *M. refringens* resistance/tolerance particularly with the objective of re‐establishing the culture of *O. edulis* on the foreshore.

## 5 CONCLUSION

In this study, we present a high‐quality genome assembly for the flat oyster *O. edulis* as a tool for further genomic studies. The analysis of tRNA, miRNA, and TEs revealed some specificities in this genome. The preliminary transcriptomic analysis made on the response of *O. edulis* to *M. refringens*, opens new research opportunities to better understand the molecular mechanisms involved in the oyster‐parasite interactions, which are not currently understood despite the highly lethal nature of this parasite. Our study provides also a valuable reference genome for further comparative genomics and population genetic analysis or to detect the signature of local adaptation and genomic re‐arrangement such as chromosome inversion as observed in several marine taxa. This genome provides a useful resource for genome‐wide studies of production traits and genomic selection which may be essential for the sustainable development of flat oyster aquaculture and restoration.

## CONFLICT OF INTEREST

The authors declare no conflicts of interest.

## Supporting information


Figures S1–S3

Figure S4

Table S1

Table S2

Table S3

Table S4

Table S5

Table S6

Table S7

## Data Availability

Raw sequence reads can be downloaded at the National Center for Biotechnology Information (NCBI) under BioProject PRJNA772088. The *Roscoff_O.edulis‐V1* genome is available under the accession JAMSGK010000000.
