# Supplementary material for: Chromosomal assembly of the flat oyster (Ostrea edulis L.) genome as a new genetic resource for aquaculture
Source: Evol Appl. 2022 Oct 10;15(11):1730–48. doi: 10.1111/eva.13462 (PMC9679248; doi:10.1111/eva.13462)
Supplement: Supplementary file 1 — Figures S1–S3 [file EVA-15-1730-s009.docx]

**Supplementary data :**


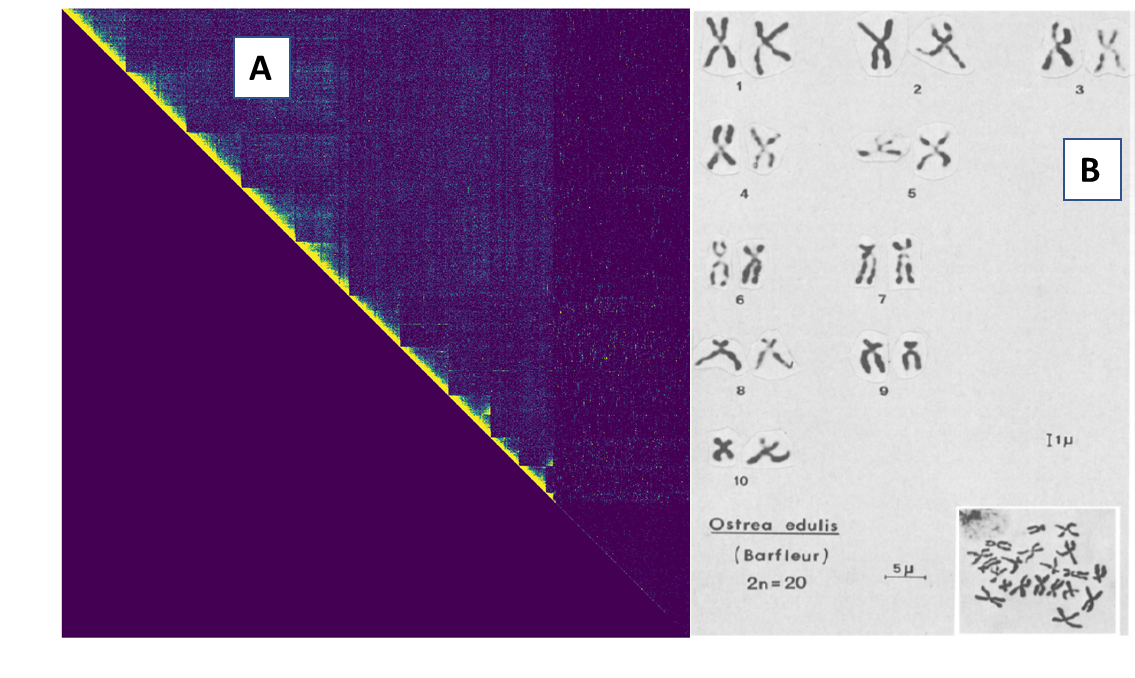


**Supplementary Figure 1** : A : The 10 main scaffolds of *Roscoff_O.edulis-V1* genome after instaGRAAL scaffolding. B : Karyotype of *O. edulis* according to Thiriot-Quiévreux & Ayraud (1970).


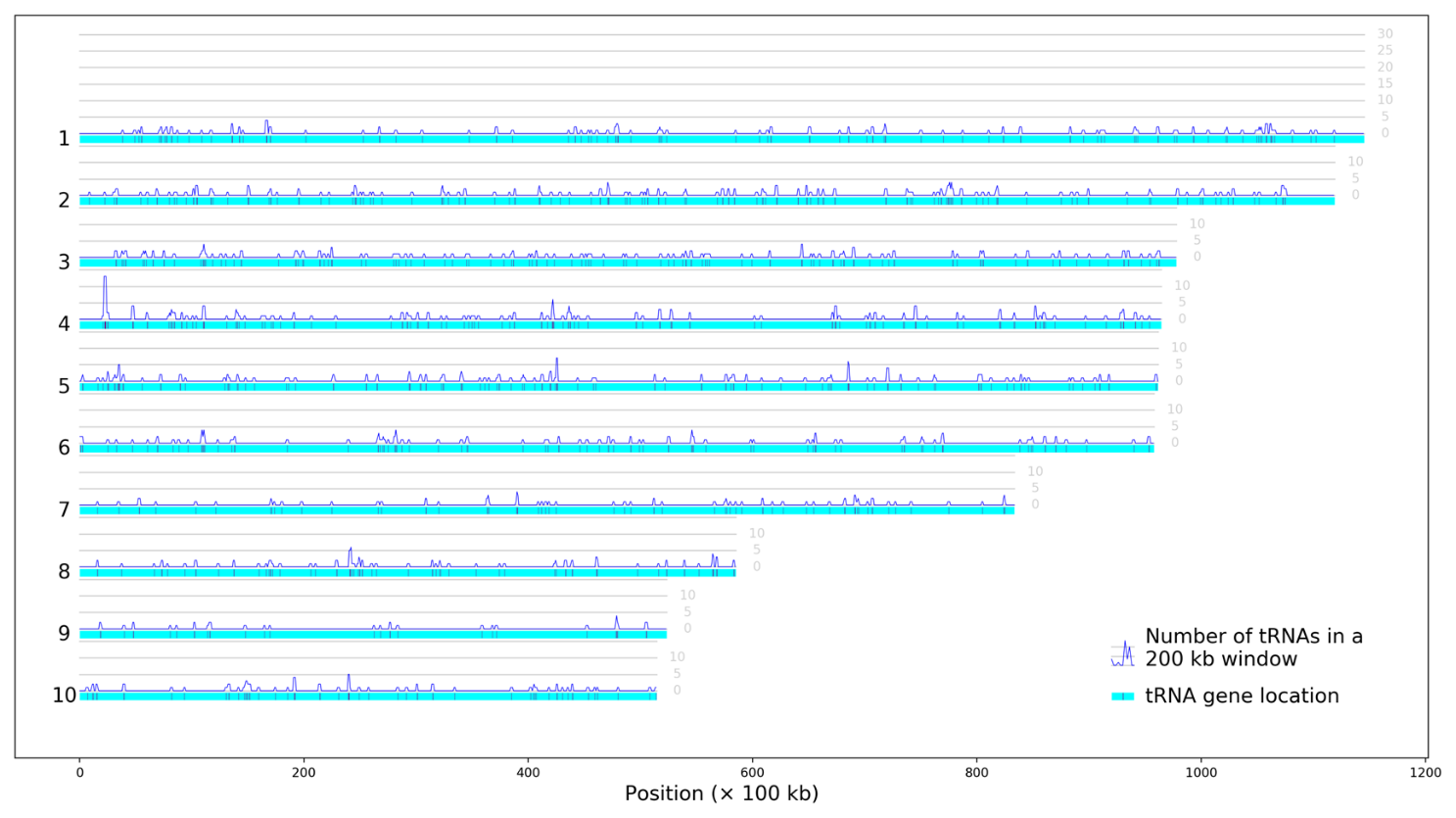


**Supplementary Figure 2** : Distribution of the RNA-Ser along the 10 chromosomes in *Roscoff_O.edulis-V1* genome


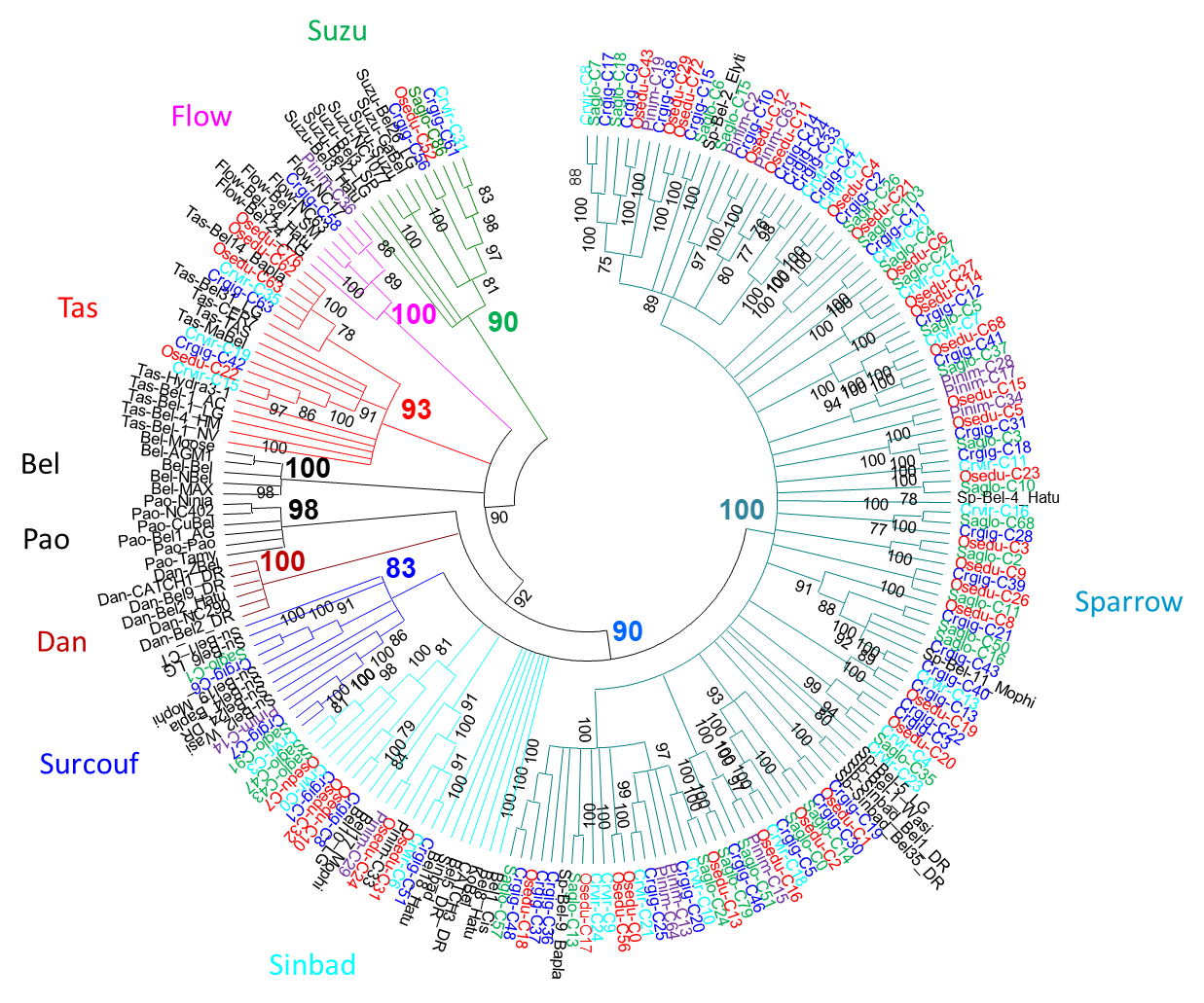


**Supplementary Figure 3** : Phylogenetic relationships of BEL/Pao retrotransposons**.** The tree is based on Neighbor-Joining analysis of RT/RNaseH domain amino acid sequences. The BEL/Pao sub-families from oysters are indicated in color (*Crassostrea gigas* in dark blue, *Crassostrea virginica* in light blue, *Ostrea edulis* in red, *Pinctada martensii* in purple and *Saccostrea glomerata* in green) as are the clades known to possess elements of molluscs. Node statistical support values (>70 %) come from non-parametric bootstrapping using 100 replicates

**Supplementary Figure 4 :**  A detailed tree representation of the clades of the Gypsy superfamily

**Supplementary Table 1** : Table 1A : identification and copy number of miRNA in the genome of *Ostrea edulis, Crassostrea. virginica and Mercenaria mercenaria*. Table 1B : distribution of the copy number for the 25 most represented miRNA in the 10 chromosomes of the *Roscoff_O.edulis-V1* genome.

**Supplementary Table 2 :** Genomic proportions of the sub-families and clades of LTR-retrotransposons detected in *Crassostrea. gigas*, *Crassostrea. virginica*, *Ostrea edulis*, *Pinctada martensii* and *Saccostrea glomerata* genomes.

**Supplementary Table 3 :** Gene ID, GOterms and enriched GOterms for Biological Process, Cellular Component and Molecular Function of genes down-regulated in *M. refringens* infected hemocytes of *O. edulis*.

**Supplementary Table 4 :** Gene ID, GOterms and enriched GOterms for Biological Process, Cellular Component and Molecular Function of genes up-regulated in *M. refringens* infected hemocytes of *O. edulis*.

**Supplementary Table 5 :** Gene ID, GOterms and enriched GOterms for Biological Process, Cellular Component and Molecular Function of genes up-regulated in *M. refringens* infected digestive gland of *O. edulis*.

**Supplementary Table 6 :** Gene ID, GOterms and enriched GOterms for Biological Process, Cellular Component and Molecular Function of genes down-regulated in *M. refringens* infected digestive gland of *O. edulis*.

**Supplementary Table 7:** Table 7.1 : normalized log2 expression of SLCs in different tissues from Ostrea edulis : the palp (Palp), mantle (MT), adductor muscle (Mus) , gills (Gill), gonads (Gon) and hemocytes (Hemo) and digestive gland (Digl), Table 7.2 : number of SLCs genes in the 10 chromosomes, Table 7.3 : number of genes per SLC family, Table 7.4 : repartition of the 7 most represented SLCs in the chromosomes.
